# Tert-Butylhydroquinone (TBHQ) Suppresses LPS- and Poly (I:C)-Induced RAW 264.7 Macrophage Activation Through Reduced NF-κB/Type 1 Interferon and Enhanced Antioxidant-Related Pathways

**DOI:** 10.3390/toxics13100883

**Published:** 2025-10-16

**Authors:** Alyssa M. Whisel, Charles D. Rice

**Affiliations:** Graduate Program in Environmental Toxicology, Department of Biological Sciences, Clemson University, Clemson, SC 29634, USA

**Keywords:** TBHQ, macrophage activation, oxidative stress, immunotoxicology, inflammation

## Abstract

The global demand for processed foods has increased reliance on synthetic phenolic antioxidants (SPAs), including tert-butylhydroquinone (TBHQ), a widely used additive to prevent lipid oxidation and extend shelf life. TBHQ is considered safe at present regulated levels; however, studies suggest potential adverse effects, including oxidative stress, genotoxicity, and impacts on immune function, raising concerns about human health and ecological risks. Herein, we investigated the immunomodulatory effects of TBHQ on RAW 264.7 murine macrophages pre-exposed to 0.1, 1, and 5 µM TBHQ and then stimulated with lipopolysaccharide (LPS) or polyinosinic-polycytidylic acid (poly I:C, PIC) to model bacterial and viral immune challenges. We then used functional assays and transcriptomic profiling to assess inflammatory responses and oxidative stress signaling. TBHQ reduced nitric oxide production and IL-10 secretion at the highest non-cytotoxic dose, and enhanced phagocytosis and IL-6 secretion at the lowest concentrations. Overall, transcriptomics revealed significant downregulation of proinflammatory pathways and induction of glutathione and xenobiotic metabolism. Pre-treatment with TBHQ increased gene transcript counts of key metabolic genes/transporters such as *Cbr3*, *Adh7*, *Gstp1/3*, *Gsta3*, *Hmox1* and *Gclm*. Following treatment with LPS or PIC several genes for classical proinflammatory chemokines and cytokines such as *Cxcl2*, *Ccl2*, *Ccl12*, *Acod1*, *Ptgs2*, *Nos2*, and *Il6* were downregulated. Genes involved in NF-κB signaling, such as *Nfkbia*, *Nfkb1*, and *Ikbke* were also downregulated. Our study suggests that the induction of Nrf2-related antioxidant pathways by TBHQ is the main driver for reduced inflammatory signaling in macrophages.

## 1. Introduction

The current world population is projected to reach 9 billion by 2050 [1]. Meeting the demands of a growing population requires large-scale food production, which is tightly regulated to ensure safety and quality [2]. Historically, food safety has focused on preventing contamination from pathogenic microorganisms; however, the concept now extends to chemical additives that influence the color, flavor, and shelf life of food products, as well as to improve organoleptic properties [2,3]. Additionally, the increasing consumption of pre-packaged and ready-to-eat foods has led to the widespread use of additives to enhance palatability and prevent spoilage [4].

Among food additives, synthetic phenolic antioxidants (SPAs), such as butylated hydroxyanisole (BHA), butylated hydroxytoluene (BHT), and tert-butylhydroquinone (TBHQ), are commonly used to prevent oxidative deterioration and extend product shelf life [5]. These synthetic antioxidants are recognized for being highly efficacious, chemically stable, and cost-effective, and they may be viewed more favorably than their natural antioxidant counterparts [6,7]. Mechanistically, SPAs function via scavenging free radicals, thereby delaying oxidation and preserving the organoleptic properties of food products [8]. Among these, TBHQ exhibits the most potent antioxidant activity and chemical stability [9,10]. It is designated as “Generally Recognized as Safe” (GRAS) by the U.S. Food and Drug Administration (FDA), with a maximum allowable concentration of 0.02% relative to the fat/oil content of the food product. The Joint FAO/WHO Expert Committee on Food Additives (JECFA) reported an Acceptable Daily Intake (ADI) of TBHQ to be 0–0.7 mg/kg body weight [11,12]. Despite regulatory parameters, recent concerns have arisen over potential excess consumption and/or overexposure, particularly since TBHQ is a primary metabolite of the carcinogen BHA, which compounds the risk of cumulative exposures [7,13]. Other applications of TBHQ can include food packaging, cosmetics and personal care products, and industrial resins, raising questions about unintended ecological and environmental exposure [14,15,16,17].

Early toxicological evaluations of TBHQ raised concerns over potential genotoxicity and dose-dependent effects [18]. The U.S. National Toxicology Program (NTP) evaluated TBHQ and it concluded that it had no carcinogenic activity in rodents following oral exposure, although higher doses were associated with reduced body weight, sex-dependent reproductive alterations, and increased nasal epithelial hyperplasia [19]. TBHQ exhibited limited genotoxicity, with negative results in the Ames test, but showed evidence of chromosomal aberrations and increased sister chromatid exchange in a metabolically active in vitro model [19]. Oral administration is the most common route of exposure TBHQ [7], and is metabolized into its quinone derivative, tert-butyl benzoquinone (TBBQ), via oxidation and conjugation before the majority of the compound is excreted through the urine [7]. More recent studies have expanded toxicity assessments to zebrafish embryos and larvae [20,21,22,23] and hepatocytes [24], linking effects to activation of the master antioxidant regulator Nrf2 [25,26,27,28,29,30]. Moreover, TBHQ has also been identified as a potential aryl hydrocarbon receptor (AHR) agonist [31].

Although TBHQ is a potent antioxidant for the food and packaging industry, in living systems, it induces oxidative stress by activating the Keap-1/Nrf2 pathways and other electrophilic mechanisms [30,32,33,34]. The effects of TBHQ on the immune system remain understudied. Existing studies have largely focused on natural killer (NK) cells [30] and T cells [25,28,29], suggesting possible effects on both innate and adaptive immunity. As a target organ, the immune system is a distributed defense network that relies on epithelial barriers and resident sentinel immune cells, such as macrophages, to detect and respond to pathogens [35]. Tissue resident macrophages are derived from circulating monocytes and are phagocytic leukocytes that play a central role in innate immunity by engulfing pathogens, clearing apoptotic cells, and releasing cytokines to orchestrate inflammation [36,37]. They also serve as antigen-presenting cells, bridging innate and adaptive immunity [38]. To our knowledge no studies to date have directly evaluated the immunotoxic potential of TBHQ in macrophages, representing a critical gap in its broader health ecological impacts.

In this study, we investigate the immunomodulatory effects of TBHQ using RAW 264.7 macrophages as an in vitro model. We tested the hypothesis that exposure to TBHQ would inhibit inflammatory signaling and this would be reflected in reduced basic macrophage functions and transcriptomic profiles. We also tested the hypothesis that pre-exposure to TBHQ treatment would induce oxidative stress through Nrf2 activation directly or contribute to inflammation-related oxidative stress and suppress inflammatory responses. Our approach was to first pre-expose macrophages to TBHQ and then stimulate with lipopolysaccharide (LPS) or polyinosinic-polycytidylic acid (poly I:C, PIC), to model Gram-negative bacterial and viral challenges, respectively, and to evaluate changes in inflammatory responses due to exposure. Second, we examined transcriptomic responses in macrophage pre-exposed to TBHQ for 4 h and compared these transcripts to cells also stimulated with LPS or PIC for 12 h following the 4 h TBHQ treatment. This experimental design was chosen to reveal the oxidative stress transcriptome at the time of activation by the model stimulants. While ongoing efforts aim to identify natural products and small-molecule pharmaceuticals that reduce inflammation by activating Nrf2 pathways under controlled conditions [39,40], the unwanted effects of environmental inducers of this pathway, like TBHQ, may suppress normal and essential inflammatory processes. The findings from this study aim to fill existing gaps in TBHQ immunotoxicity and contribute to a more comprehensive understanding of TBHQ safety and risk assessment.

## 2. Methods and Materials

### 2.1. Cell Culture

RAW 264.7 murine macrophages (ATCC TIB-71) at passage three were cultured in T-75 flasks (FB012937, Fisher Scientific, Pittsburg, PA, USA) at 37 °C with 5% CO_2_. Cells were grown in Dulbecco’s Modified Eagles’s Medium (DMEM; 15-018-CV, Corning, Manassas, VA, USA) supplemented with 10% bovine fetal bovine serum (FBS; 35-010-CV, Corning, Woodland, CA, USA), 1% nonessential amino acids (NEAA 100×; 11-140-050, Gibco, Carlsbad, CA, USA), 1% sodium bicarbonate (61-065-RO, Cellgro, Manassas, VA, USA), 1% penicillin-streptomycin-glutamine solution (P/S-G 100×; 10-378-016, Gibco, Carlsbad, CA, USA), 20 µg/mL gentamycin sulfate (BP918-1, Fisher BioReagents, Fair Lawn, NJ, USA), and 10 µg/mL nystatin (BP2949-5, Fisher Scientific, Fair Lawn, NJ, USA). Complete media was supplemented with L-glutamine (G-5763, Sigma, St. Louis, MO, USA) for a final concentration of 4 mM.

### 2.2. Chemicals/Treatments

Tert-butylhydroquinone (TBHQ; 150820050, Thermo Scientific Chemicals, Fair Lawn, NJ, USA) was diluted in anhydrous DMSO (276855-100ML, Sigma-Aldrich, St. Louis, MO, USA) to a stock solution of 10^−2^ M and stored at −20 °C 1 week prior to use. Lipopolysaccharide (LPS) from *Escherichia coli* O127:B8 (L3129-10MG, Sigma-Aldrich, St. Louis, MO, USA) was diluted in water to a stock solution of 1 mg/mL and stored at −20 °C 7 2 weeks prior to use. Polyinosinic-polycytidylic acid (poly I:C, PIC) sodium salt (61401S, Cell Signaling Technology, Danvers, MA, USA) was diluted in sterile water to a stock solution of 5 mg/mL and stored at −20 °C 2 weeks prior to use. For experiments using microtiter assays, RAW264.7 macrophages were seeded into 96-well plates (3595, Costar, Kennebunk, ME, USA) at 10^5^ cells per well in 100 µL of phenol-free DMEM media (17-205-CV, Corning, Manassas, VA, USA). For assays using larger culture approaches, RAW264.7 cells were seeded in twelve 6-well plates (353846, Corning, New York, NY, USA) at 2 × 10^5^ cells per well in 3 mL of complete media.

### 2.3. Initial Dose-Dependent Screening Assays to Determine TBHQ Cytotoxicity

Cellular respiration as an indication of cytotoxicity was measured by the MTT (3-[4,5-dimethylthiazol-2-yl]-2,5-diphenyltetrazolium bromide) assay using standard protocols [41]. Macrophages were plated into 96 well Costar plates at 10^5^ cells per well in 100 μL of DMEM media. After 4 h of incubation at 37 °C for adherence, TBHQ and DMSO carrier control (0.01% final) were added in triplicate over serial dilutions beginning with 10 μM per well in a total volume of 200 μL, and the plates incubated for 24 h followed by processing for optical density.

A colorimetric inducible nitric oxide synthase (iNOS) assay was used to screen for dose-dependent effects of TBHQ treatment on basic macrophage functions to compare with the cytotoxicity assays. Procedures for the assay followed standard protocols [42]. Cells were co-treated with TBHQ and the model stimulants LPS and PIC using the following approaches. Wells containing cells were pre-treated with serial dilutions of 10 µM TBHQ in a final volume of 200 µL for a 4 h period based on studies by others [43,44], followed by 22 µL of LPS or PIC at a final concentration of 0.1 µg/mL or 20 µg/mL PIC, respectively, for 20 h. Cytokine secretion and a phagocytosis assay were also used to determine dose-dependent toxicities, and based on the MTT and iNOS assay, these two functions were examined at concentrations of 0.1, 1, or 5 µM TBHQ.

Mouse IL-6 (431304) and IL-10 (431414) ELISA MAX^TM^ Deluxe Sets (BioLegend, San Diego, CA, USA) were used to quantify cytokine secretions in macrophage supernatants after 24 h treatment using the manufacturer’s protocol. Cytokine concentrations were interpolated from standard curves and expressed relative to the positive controls (LPS- or PIC-stimulated macrophages). Macrophage phagocytosis of Fluoresbrite™ carboxylate YG 1.5 µm microspheres (#09719, Polysciences, Warrington, PA, USA) was assessed after the above 24 h treatment as previously described [41].

Subsequent studies for RNA sequencing and related protein validation were carried out after 12 h exposure using 0 and 5 µM TBHQ.

### 2.4. RNA Isolation for Bulk RNAseq Analysis

For RNA sequencing experiments, RAW 264.7 macrophages were seeded in twelve 6-well plates (353846, Corning, New York, NY, USA) at a density of 2 × 10^5^ cells per well in 3 mL of complete medium for 4 h for adherence beginning at noon. The experimental design included six treatment groups with three biological replicates. In the control group macrophages received no further treatment. In the second group macrophages were treated at 4:00 p.m. with 5 µM TBHQ and incubated for 4 h, and this group served as the baseline for transcriptomic profile analysis at the time of further treatments. The remaining four treatment groups were stimulated with either 0.1 µg/mL LPS (group 3) or 20 µg/mL PIC (group 4), or 5 µM TBHQ for 4 h and either LPS (group 5) or PIC (group 6) for an additional 12 h. Following treatment, media was removed from wells and cells were lysed in 1 mL TRI Reagent (R2051A, Zymo Research, Irvine, CA, USA). Lysates were immediately stored at −80 °C for 5 days prior to RNA extraction.

### 2.5. Sample Preparation and mRNA Sequencing

Total RNA was isolated using the Direct-zol RNA Miniprep Kit (R2051A, Zymo Research, Irvine, CA, USA) according to the manufacturer’s protocol. The samples were thawed at room temperature prior to processing. Briefly, an equal volume of 100% ethanol was added to each lysate in TRI Reagent, mixed thoroughly, and applied to the spin column. After centrifugation, the flow-through was discarded, and the bound RNA was treated with DNase and washed before elution in DNase/RNase-free water.

From this point all samples were processed by Novogene Corporation, Inc. (Sacramento, CA, USA). Messenger RNA (mRNA) was purified via poly(A)-tail enrichment, and strand-specific libraries were constructed. Library concentration was quantified via Qubit and real-time PCR, and fragment size distribution was assessed by Agilent Bioanalyzer (Agilent Technologies, Inc., Santa Clara, CA, USA). Quantified libraries were then sequenced on an Illumina NovaSeq X Plus (Illumina, Inc., San Diego, CA, USA) using a paired-end 150 run (2 × 150 bases). Sequencing depth was approximately 6 GB per sample.

### 2.6. RNAseq Data Analysis

Initial quality control, read alignment to the *Mus musculus* reference genome (GRCm38/mm39), transcript assembly, and normalization using fragments per kilobase of transcript per million mapped reads (FPKM) were performed by Novogene Corporation, Inc. (Sacramento, CA, USA).

Principal component analysis (PCA) was used to assess sample clustering and variability. Differential gene expression (DGE) analysis was conducted using the DESeq2 package in R on raw count data. Unless otherwise specified, genes were considered significantly differentially expressed if both the *p*-value and false discovery rate (FDR) were <0.01 and the absolute log2 fold change (|log_2_FC|) exceeded 2.

Treatment-specific gene expression profiles were evaluated via two-way Venn diagrams generated between treatment groups (LPS vs. LPS + TBHQ and PIC vs. PIC + TBHQ), based on DGE thresholds. To identify biologically relevant gene expression patterns, Kyoto Encyclopedia of Genes and Genomes (KEGG) pathway enrichment analysis was performed via kegga package. Additional data visualization was conducted in R, where dot plots of enriched KEGG pathways were constructed using ggplot2 and dplyr. Heatmaps displaying gene-level expression changes were created using the Morpheus web interface (Broad Institute, Cambridge, MA, USA; https://software.broadinstitute.org/morpheus) URL (last accessed on 9 July 2025). All bar graphs depicting either total DGE numbers or normalized FPKM expression values for specific genes of interest were composed in GraphPad Prism 10.

### 2.7. Western Blotting for Protein Expression in Comparison to RNAseq Data

RAW 264.7 cells were seeded in twelve 6-well plates (353846, Corning, New York, NY, USA) at 2 × 10^5^ cells per well in 3 mL of complete media. Cells were treated as described for RNAseq experiments. After treatment, cells were harvested by scraping and centrifuged at 1160 RPM for 10 min. Pellets were lysed in 1× NP-40 buffer (#FNN0021, Invitrogen, Vienna, Austria) containing protease inhibitors Halt Protease and Phosphatase Inhibitor Cocktail (100×; #78440, Thermo Scientific, Rockford, IL, USA), 200 µM 4-(2-Aminoethyl) benzenesulfonylfluoride (AEBSF HCl; #AG-CR1-3610-M250 AdipoGen Life Sciences, San Diego, CA, USA), and 200 µM phenylmethylsulfonyl fluoride (#800263, MP Chemicals, CA, USA). Lysates were vortexed, sonicated, and protein concentration was quantified via BCA assay. Lysates were diluted to a common protein concentration, and sample buffer (6×; J61337.AC, Thermo Scientific, Ward Hill, MA, USA) was added, and samples were boiled for 5 min.

Thirty-five µg of protein was loaded per lane on pre-cast 4–20% SDS-PAGE gels (456–8094, BioRad Laboratories, Hercules, CA, USA) and electrophoresed at 200 V. Proteins were transferred to nitrocellulose membranes (10600003, Cytiva, Darmstadt, Germany) at 100 V for 1 h. Membranes were then blocked overnight at 4 °C in 3% BSA (IgG-free, protease free. #001-000-4162, Jackson ImmunoResearch labs, West Grove, PA, USA). Primary antibodies against inducible nitric oxide synthase (iNOS; 610328, BD Transduction Laboratories, Franklin Lakes, NJ, USA), cyclooxygenase 2 (COX-2; 160126, Cayman Chemical, Ann Arbor, MI, USA), and β-actin (66009-1-Ig, Proteintech, Rosemont, IL, USA) were added at recommended dilutions in PBS-TW20. A monoclonal antibody was generated in house against the synthetic peptide DTFYGHWRKPLSQE mapping to residues 404–417 of mouse ACOD1 (accession # NP_032418.1) and used as confluent hybridoma supernatants diluted 1:5 in PBS-TW20. The details of immunogen selection, immunizations, hybridoma production and cloning, and antibody characterization are available in Supplemental (Materials and Methods, Table S1) based on previous procedures [45,46]. The synthetic peptide and hybridoma/mAb (mAb AW-12) and quantitative antigen-binding data are under the intellectual property management of the Clemson University Research Foundation, and the three CDRs have been submitted as an invention disclosure for further patenting and commercialization.

Membranes were incubated with antibodies at room temperature for 2 h while gently rocking. Membranes were washed with PBS-TW20 3 × 10 min, followed by secondary antibody incubation with rabbit anti-mouse IgG-AP diluted 1:2000 in PBS-TW20 (#AB6729, Abcam, Waltham, MA, USA) or goat anti-rabbit IgG-AP (ThermoFisher, #A-10931) diluted 1:2000 for 1.5 h while gently rocking. After multiple washings with PBS-TW20 the blots received NBT-BCIP developing solution (Thermo-Scientific™: PI34042). Blots were visualized via ChemiDoc™ MP Imaging System (BioRad) and band densities were quantified using ImageJ (Fiji version) software [47].

### 2.8. Statistical Analysis

All statistical analyses were performed using GraphPad Prism 10 (GraphPad Software, San Diego, CA, USA). For percentage data, values were transformed prior to analysis. Shapiro–Wilk tests for homogeneity of variances and Brown-Forsythe tests for normality were used to validate the use of parametric statistics. One-way analysis of variance (ANOVA) was then used, followed by a Tukey test to compare treatment groups. Statistical significance thresholds were preset with an α = 0.05. All the assays were conducted with three biological replicates. Statistical analyzes related to transcriptomic data, including differential gene expression and enrichment analyses, were conducted separately as described in data analysis.

## 3. Results

### 3.1. Cytotoxicity Assays

**Figure 1:**
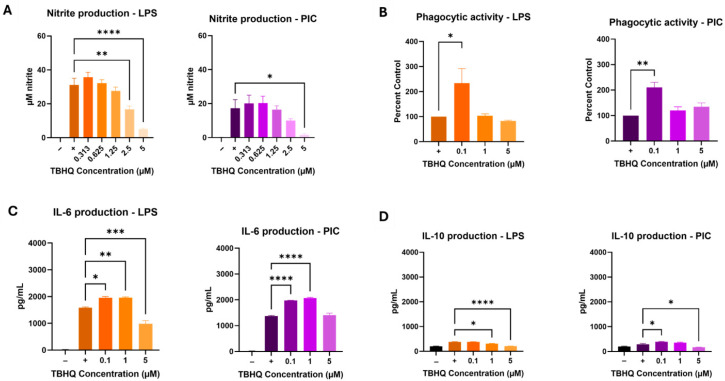
RAW 264.7 macrophages were treated with media only, or dilutions of TBHQ for 4 h prior to the addition of 0.1 µg/mL of LPS or 20 µg/mL PIC for 24 h. (**A**) Nitrite secretion measured directly in the cell supernatants via the Griess Reagent. (**B**) Ability of macrophages to phagocytize 1.5 µm fluorescent microspheres. (**C**) IL-6 cytokine secretion measured by ELISA. (**D**) IL-10 cytokine secretion measured by ELISA. All experiments were performed in triplicate (*n* = 3). Data represents the mean ± SE. Statistical significance is represented by * (*p* ≤ 0.05), ** (*p* ≤ 0.01), *** (*p* ≤ 0.001), and **** (*p* ≤ 0.0001).

TBHQ at levels of 5 µM and below was not cytotoxic at 24 h (Supplemental Data, Figure S1). Initial iNOS activity assays to determine appropriate ranges of treatment levels in biological assays used serial dilutions of 5 µM, which resulted in reduced activities induced by LPS and PIC over a 24 h period (Figure 1A). Under TBHQ treatment and LPS stimulation, 2.5 µM and 5 µM doses reduced iNOS activity compared to untreated cells. However, under TBHQ treatment and PIC stimulation, only 5 µM dose reduced iNOS activity. Notably, LPS stimulation produced a larger output of nitrite compared to PIC in RAW 264.7 cells under the conditions of this study. Subsequent experiments to further determine effects of TBHQ on basic macrophage functions used fixed levels of 0, 0.1, 1, and 5 µM.

### 3.2. TBHQ Suppresses Proinflammatory Properties of Activated RAW 264.7 Macrophages

Macrophage phagocytosis was also tested, as this is one of the key innate immune functions of the cell type. Macrophages showed increased phagocytic activity at the low (0.1 µM) dose of TBHQ under both LPS- and PIC-stimulated conditions after 24 h of culture (Figure 1B). Exposure to 1 µM and 5 µM did not significantly impact phagocytic activity in activated cells.

Collected supernatants were screened for proinflammatory cytokine secretion using commercially available enzyme-linked immunosorbent assays (ELISAs) against IL-6 (Figure 1C), a classical pro-inflammatory product, and IL-10 (Figure 1D), a classical anti-inflammatory product. LPS-stimulated IL-6 levels increased in cells exposed to 0.1 µM and 1 µM concentrations of TBHQ, but levels were suppressed in cells treated with 5 µM. Similar results were noted when stimulated with PIC, however the highest concentration of TBHQ did not reduce IL-6 expression. On the other hand, anti-inflammatory signaling by IL-10 was minimal. Under LPS simulated conditions, 1 µM and 5 µM doses of TBHQ reduced IL-10 secretion, whereas PIC stimulated conditions saw a slight increase at the 0.1 µM TBHQ before decreasing in 5 µM-treated cells.

### 3.3. LPS- and PIC-Stimulated Macrophages Show Reduced Differential Gene Expression When Exposed to TBHQ

**Figure 2:**
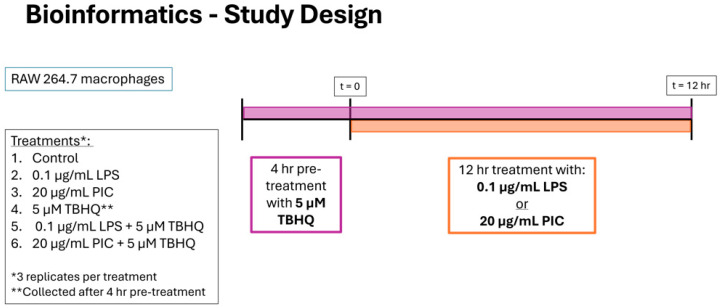
Experimental timeline and study design for bioinformatics. RAW 264.7 cells were stimulated with (1) media only, (2) 0.1 µg/mL of LPS or (3) 20 µg/mL PIC for 12 h, with designated treatment groups being pre-exposed to (4) 5 µM TBHQ for 4 h, (5) TBHQ for 4 h, and LPS for an additional 12 h, or (6) TBHQ for 4 h, and PIC for an additional 12 h. Cells were then lysed with Trizol before total RNA was collected and enriched for sequencing. All experiments were performed in triplicate (*n* = 3).

**Figure 3:**
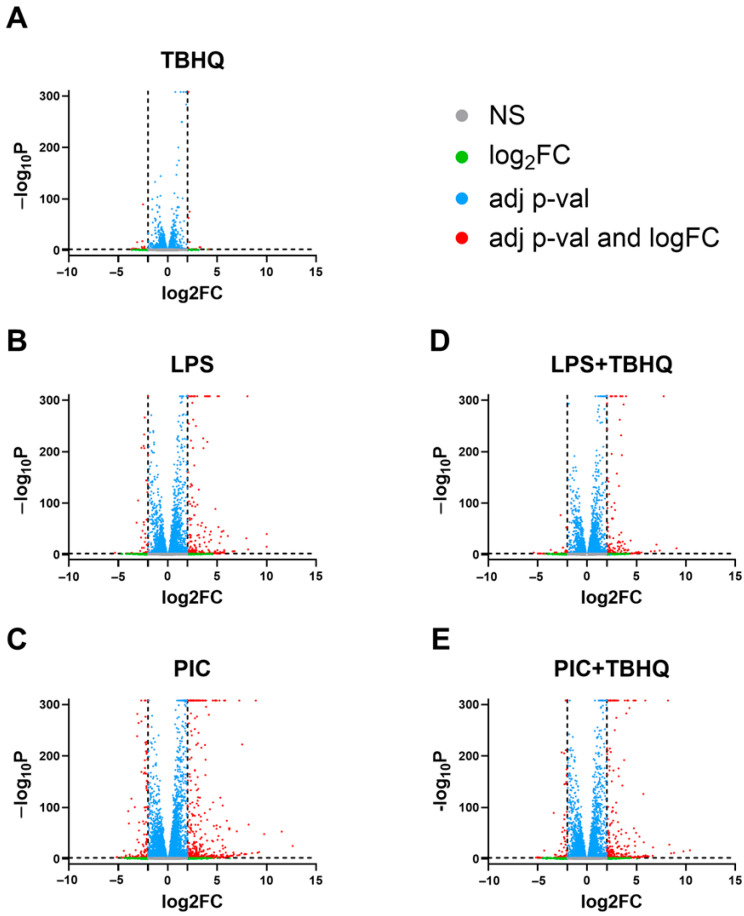
Volcano plot of differentially expressed genes in (**A**) macrophages pretreated for 4 h with 5 µM TBHQ, (**B**) macrophages treated for 12 h with 0.1 µg/mL of LPS, (**C**) macrophages treated for 12 h with 20 µg/mL PIC, (**D**) macrophages pretreated for 4 h with 5 µM TBHQ and treated for 12 h with 0.1 µg/mL of LPS, and (**E**) macrophages pretreated 4 h with 5 µM TBHQ and treated for 12 h with 20 µg/mL PIC. All genes are shown as dots. Significantly differentially expressed genes (*p* value ≤ 0.01) are colored above the horizontal line as blue or red. Genes with a differential expression of >|2| are shown in red to the left and right of the vertical lines.

Herein we examined transcriptomic responses in macrophages pre-exposed to TBHQ for 4 h and compared these transcripts to cells also stimulated with LPS or PIC for 12 h following TBHQ treatment (Figure 2). Untreated cells served as the control. Biological replicates were tightly grouped in PCA (Supplemental Figure S2). Cells treated with TBHQ for 4 h showed minimal differential gene expression (Figure 3A). Conversely, cells stimulated with LPS or PIC showed high levels of differential gene expression, with most significant genes being upregulated (Figure 3B,C). When pre-treated with TBHQ, stimulated macrophages showed a marked decrease in differential gene expression (Figure 3D,E).

**Figure 4:**
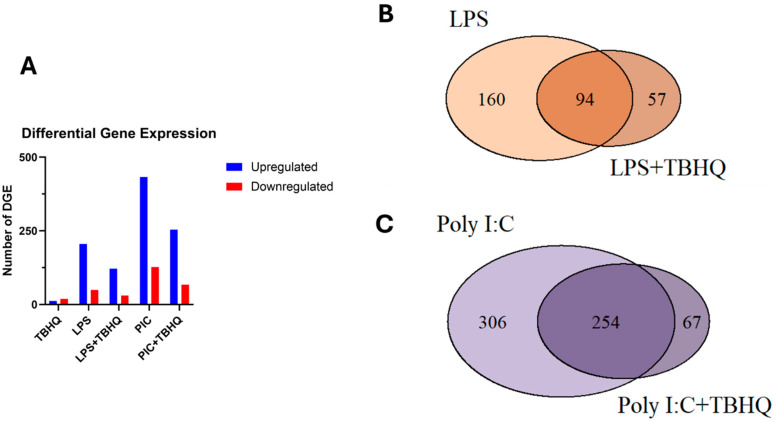
(**A**) Total number of up- and down-regulated differentially expressed genes that were significant (*p* value ≤ 0.01 and log2FC > |2|). Venn diagram of up-regulated differentially expressed genes in LPS versus LPS+TBHQ (**B**) treated conditions and PIC versus PIC + TBHQ treated conditions (**C**).

Differential gene expression (DGE) analysis of treated vs. control macrophages revealed significantly more upregulated genes compared to those that were downregulated (Figure 4A). Total DGE decreases by 59% and 57% in LPS- and PIC-stimulated conditions when pre-treated with TBHQ, respectively. Regarding the treatment conditions, expression of multiple genes overlapped between stimulated populations and those pre-treated with TBHQ (Figure 4B,C). However, a small subset of genes, 57 and 67 in “LPS+TBHQ” and “PIC+TBHQ”, respectively, remain distinct to TBHQ treatment.

### 3.4. Inflammatory and Cellular Metabolic Profiles Differ Between Treated Macrophages

**Figure 5:**
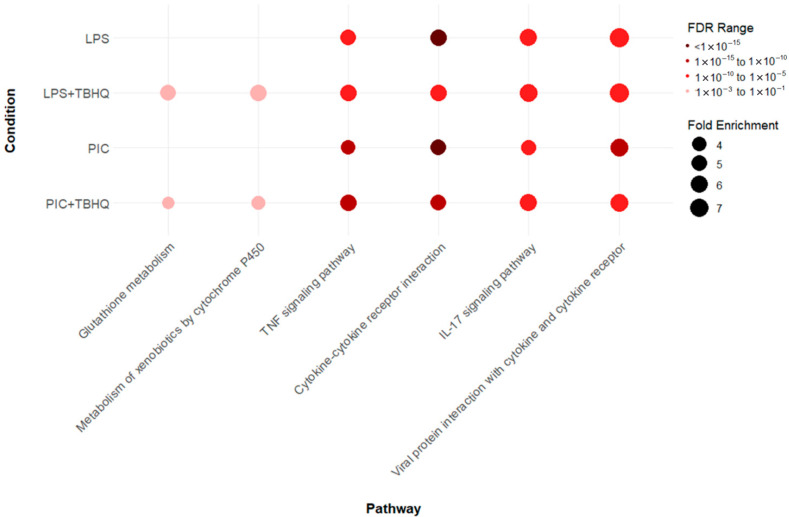
Dot plot of select Kyoto Encyclopedia of Genes and Genomes (KEGG) pathways that were up regulated in LPS- or PIC-stimulated conditions, with or without 4 h pretreatment with TBHQ. All pathways are statistically significant (FDR ≤ 0.01), and the color of the dots represent the FDR value, where darker reds are more statistically significant. The size of the dot represents the fold enrichment, where larger dots are proportional to a larger fold-enrichment value.

KEGG pathway analysis revealed 74 cumulative upregulated pathways in at least one treatment condition (Supplemental Figure S3). However, two distinct enrichments were observed within the data (Figure 5). First, inflammatory pathways TNF signaling pathway (mmu04668), cytokine-cytokine receptor interaction (mmu04060), IL-17 signaling pathway (mmu04657), and viral protein interaction with cytokine and cytokine receptor (mmu04061) were upregulated across all treatment (LPS, PIC, LPS + TBHQ, PIC + TBHQ) conditions. When cells were pre-treated with TBHQ, inflammatory pathways both decreased in fold enrichment and false discovery rate (FDR). Additionally, the number of upregulated shared genes between inflammatory KEGG pathways also decreased in the presence of TBHQ. Secondly, cells pre-treated with TBHQ were the only to show upregulated cellular metabolism pathways, glutathione metabolism (mmu00480) and metabolism of xenobiotics by cytochrome P450 (mmu00980) (Supplemental Figure S4).

**Figure 6:**
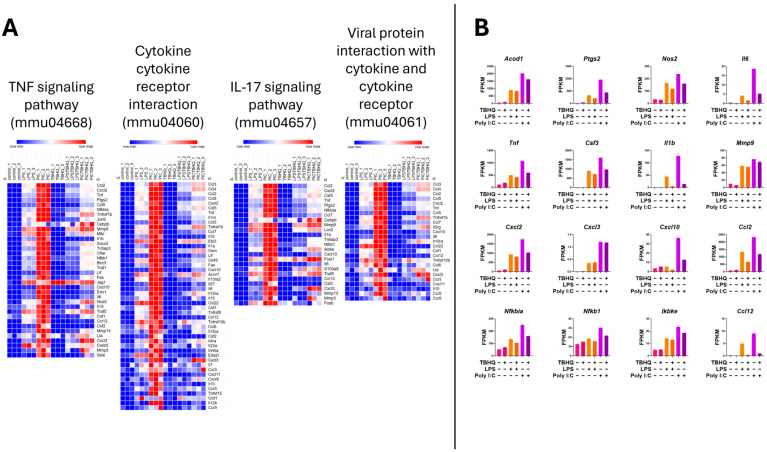
Heatmap of normalized gene counts for up-regulated genes from KEGG pathway analysis shown in Figure 5. (**A**) The KEGG pathways associated with inflammation, and (**B**) normalized gene counts (fragments per kilobase of transcript per million mapped reads, FPKM) from select genes within inflammatory KEGG pathways. Each bar represents a treatment condition (*n* = 3).

**Figure 7:**
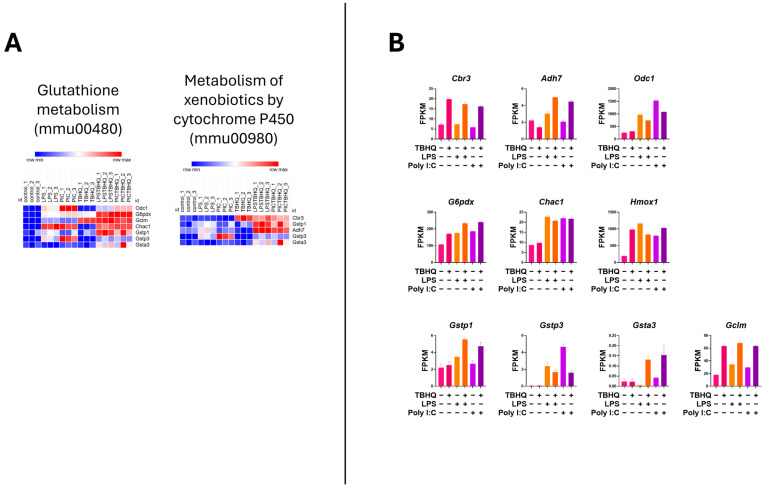
Heatmap of normalized gene counts for up-regulated genes from KEGG pathway analysis shown in Figure 5. (**A**) KEGG pathways associated with cellular metabolism, and (**B**) normalized gene counts (fragments per kilobase of transcript per million mapped reads, FPKM) from selected genes within cellular metabolism KEGG pathways. Each bar represents a treatment condition (*n* = 3).

Heatmap analysis of the upregulated genes within the inflammatory KEGG pathways showed higher mRNA expression when stimulated with PIC compared to LPS stimulation (Figure 6A). Again, when in the presence of TBHQ, gene expression dramatically decreased. Select key genes involved in macrophage inflammation are impacted, where gene-transcript counts increase following LPS and PIC and are slightly reduced when pre-treated with TBHQ (Figure 6B). These include classical proinflammatory chemokines and cytokines such as *Cxcl2*, *Ccl2*, *Ccl12*, *Acod1*, *Ptgs2*, *Nos2*, and *Il6*. Genes involved in NF-κB signaling, such as *Nfkbia*, *Nfkb1*, and *Ikbke*, follow the same trends. Of note, none of the pro-inflammatory genes were increased by pre-treatment with TBHQ alone. In contrast, heatmaps depicting the upregulated genes involved in cellular metabolic KEGG pathways are induced in the presence of TBHQ (Figure 7A). This can be more clearly observed when examining the normalized gene counts individually, where pre-treatment of TBHQ increased gene transcript counts of key metabolic genes/transporters such as *Cbr3*, *Adh7*, *Gstp1/3*, *Gsta3*, *Hmox1* and *Gclm* prior to PIC or LPS stimulation of the cells (Figure 7B).

**Figure 8:**
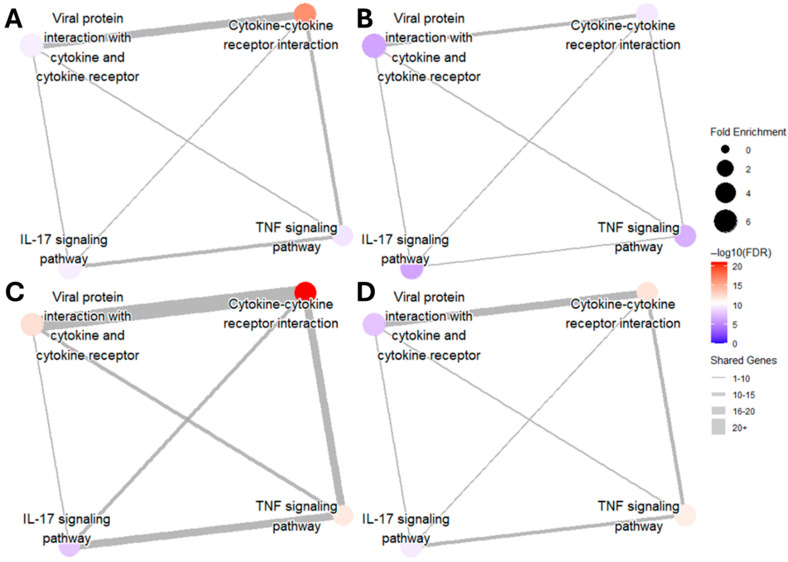
Network map of upregulated inflammatory KEGG pathways in (**A**) macrophages treated with 0.1 µg/mL of LPS for 12 h, (**B**) macrophages pretreated for 4 h with 5 µM TBHQ and then 0.1 µg/mL of LPS for 12 h, (**C**) macrophages treated with 20 µg/mL PIC for 12 h, and (**D**) macrophages pretreated with 5 µM TBHQ for 4 h and then 20 µg/mL PIC for 12 h. The size of the dots represents the fold enrichment of the labeled pathway. The color of the node represents the statistical significance of the pathway (all FDR values < 0.01). The width of the edges represents the number of shared genes between pathways.

DGE analysis followed a similar pattern as gene transcript counts (Table 1). When compared to the control condition, genes associated with inflammation showed the highest upregulation in LPS and PIC stimulation alone, with the same differential comparison being attenuated in the presence of TBHQ. DGE of cellular metabolism genes, on the other hand, shows more consistent significance when pre-treated with TBHQ. Network map analysis of KEGG pathway enrichment revealed distinct connectivity patterns across treatments (Figure 8). In all conditions, the strongest overlap of differentially expressed genes was observed between the pathways “Viral protein interaction with cytokine and cytokine receptor” and “Cytokine–cytokine receptor interaction,” as indicated by the thickest edges. Compared to their respective stimulatory controls, pretreatment with TBHQ followed by treatment with either LPS (Figure 8B) or PIC (Figure 8D) reduced overall network connectivity, fold enrichment of individual pathways, and statistical significance (smaller, lighter-colored nodes with fewer connections). Notably, macrophages stimulated with PIC (Figure 8C,D) exhibited greater pathway connectivity and enrichment than those stimulated with LPS (Figure 8A,B), suggesting a more robust transcriptional response to PIC.

### 3.5. TBHQ Attenuates Inflammatory Protein Expression and Nitric Oxide Production at 12 H

**Figure 9:**
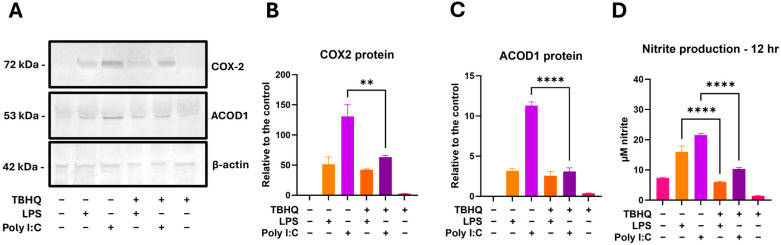
RAW 264.7 cells were pretreated with 5 µM TBHQ for 4 h, then stimulated with 0.1 µg/mL of LPS or 20 µg/mL PIC for 12 h. (**A**) Cell lysates were subjected to SDS-PAGE and immunoblotting. Representative immunoblots showing COX-2, ACOD1, and β-actin as the loading control. Western blot analysis of (**B**) COX-2 and (**C**) ACOD1. Data represents the mean band densities ± SE. (**D**) Nitrite production measured in cell supernatants via the Griess Reagent. Data represents the mean ± SE. All experiments were performed in triplicate (*n* = 3). Statistical significance is represented by ** (*p* ≤ 0.01) and **** (*p* ≤ 0.0001).

Select markers of immunomodulation were further tested at the 12 h post stimulation with LPS or PIC, following the same treatment scheme as the bioinformatics to compare transcript data with protein expression. Western blotting showed inflammatory protein COX-2 to have the largest induction in LPS and PIC alone treatments (Figure 9A,B). When cells were pretreated with TBHQ, both stimulated conditions were associated with less COX-2 protein expression. ACOD1, a key product of macrophage activation immunometabolism, followed the same trend as COX-2 (Figure 9A,C). Nitrite production measured via Griess Reagent was also observed at the 12 h timepoint, once again following the same trend, in which TBHQ pre-treatment reduced the level of nitrite measured in the macrophage supernatants (Figure 9D).

## 4. Discussion

Tert-Butylhydroquinone (TBHQ) is considered a classical inducer of oxidative stress in a variety of cell types, tissues, and organs, and activates Nrf2 by altering the thiol groups of cysteine-273 and cysteine-288 on the Keap1 protein, thereby inhibiting the repression of Nrf2 by Keap1 [48]. At least in vitro, Nrf2 activation by TBHQ is often reported at levels as high as 50–100 µM for up to 4 h [43]. However, in this study, we found that levels above 5 µM were cytotoxic to RAW 264.7 macrophages; therefore, we confined the study to this level and below, which is consistent with previous studies using T-cells and NK cells. Part of our study examined the expression of Nrf2 protein in macrophage lysates by Western blotting at the end of exposures to TBHQ, LPS, or PIC treatment, or a combination of these. Nrf2 protein was not detected, which would be expected since Nrf2 activation occurs within minutes post-exposure in immune cells [28]. The observation by others that whole cell lysate Nrf2 expression increases within a few hours after exposure to oxidative stress is likely due to de novo synthesis and transport back to the cytosol [49,50], depending on the model Nrf2 activator. After translocation to the nucleus, Nrf2, along with s-Maf, binds to the antioxidant response element (ARE) and initiates the transcription of a battery of antioxidant proteins that are present hours post-treatment [48,51]. Similarly, NF-κB and IRF activation in stimulated macrophages occurs within minutes of exposure to model stimulants like LPS and PIC used in our study [52]. However, down-stream proinflammatory products (e.g., iNOS, COX-2, ACOD1, IL-6) can be measured hours after activation.

Our in vitro model for macrophage activation is well characterized and commonly used to screen chemical compounds for their ability to modulate macrophage activation. LPS signals through both MyD88- and TRIF-dependent arms of TLR4, triggering NF-κB and type I interferon pathways [53]. PIC primarily engages TLR3 (TRIF-dependent) but may also activate cytosolic RNA sensors (e.g., MDA5, RIG-I) [54,55], leading to robust interferon-stimulated gene (ISG) expression [54]. TBHQ had similar effects on both stimuli, reducing function and inflammatory gene expression while enhancing antioxidant/metabolic pathways, but with differing magnitudes. LPS induced greater nitrite accumulation than PIC; however, mRNA transcripts showed the opposite trend (e.g., *Nos2*). Notably, the impact of TBHQ under viral-mimetic (PIC) conditions needs more exploration. Prior oxidative stress-related studies using PIC as the activator in RAW 264.7 macrophages have primarily focused on phytochemical modulators such as panaxadiol [56] and baicalein [57]. However, to our knowledge, this is the first study to evaluate TBHQ in that context. These findings broaden TBHQ’s toxicological relevance and suggest that it may influence antiviral immune responses.

RNA-sequencing at 12 h of activation following pretreatment with TBHQ revealed two dominant KEGG enrichments: (1) classical inflammatory gene transcripts (e.g., *Nos2*, *Il6*, *Tnf*, *Il1b*, *Cxcl10*) remained inducible but were downshifted in amplitude by TBHQ, and (2) glutathione-associated redox and metabolic pathways were selectively upregulated in TBHQ-treated cells. Notably, TBHQ pretreatment downregulated key NF-κB-regulated genes and interferon-stimulated transcripts, indicating suppression of both canonical inflammatory and antiviral transcriptional programs. This graded suppression, rather than binary on/off regulation, parallels the partial reduction in the NO output and may further support the concept of functional reprogramming by NO [58], at least in mice, as human macrophages are much less responsive when it comes to NO production [59]. Significantly, inflammatory signaling transcription profiles at 12 h post-stimulation correlated well with the expression of COX-2 protein, iNOS activity, IL-6 and IL-10 secretion, and phagocytosis activity at 24 h. This was also observed in iNOS activity and in the expression of COX-2 and ACOD1 proteins at 12 h post-stimulation. These correlations were evident in both the number of gene transcripts and DGE following different treatments. To our knowledge, this is the first study to look at the transcriptomic level impacts of TBHQ on RAW 264.7 macrophages. One previous study examined the impact of a major oxidative metabolite of TBHQ, tert-butylquinone (TBBQ), and reported a ROS-induced mitochondrial apoptosis [60].

The observation that TBHQ treatment of cells prior to stimulation with LPS or PIC was associated with enrichment for pathways linked to glutathione biosynthesis/turnover, NAD(P)H oxidoreductases, pentose phosphate pathway (PPP) flux, and mitochondrial remodeling/biogenesis (e.g., *Hmox1*, *Gclc*, *Gclm*, *Txnrd1*) suggest activation of Nrf2/ARE-driven cytoprotective programs. For example, elevated PPP activity regenerates NADPH, supporting both antioxidant defense and biosynthesis; mitochondrial adjustments could rebalance ATP production and redox poise, potentially constraining energetically costly NO synthesis, and provide reducing equivalents for biosynthesis in general [51]. Overall, our gene expression profile indicates an upregulated pro-glutathione pathway, the lack of which could reverse cytoprotective mechanisms.

While not examined specifically in this study, the effects of TBHQ on broader immunometabolism rewiring as a component of suppressed inflammatory profiles, including the consequences of altered ACOD1 expression, should be examined in future studies. Of note, is the absence of *Acod1* expression (up or downregulated) in any of the KEGG pathways enriched in our RNAseq data. This is surprising since ACOD1, and it is metabolic product itaconate, are now considered a hallmark biomarker of, and key player in macrophage activation (M1 phenotype) and immunometabolism [61,62]. It is possible that KEGG pathway algorithms have not been updated to include *Acod1* as part of multiple genes involved in classical inflammatory responses. Notably, using mAb AW-12 developed in this study, we show an induction of ACOD1 protein due to stimulation with both LPS and PIC, and the reduction in expression associated with pre-exposure to TBHQ. Clearly, more research is needed to determine how, or if, exposure to antioxidants (whether natural or synthetic) affects immunometabolism as a mechanism of action in immunotoxicological studies.

In understanding the implications of our study, sampling at 12 h likely captured the rising or plateau phase of TBHQ-driven antioxidant processes rather than the initiating oxidative stress triggers, such as Nrf2 activation. Earlier sampling (≤6 h) in future work should reveal the onset of electrophile sensing and transcription factor activation, and elements of Nrf2/NF-κB crosstalk. Our in vitro model is intended to simulate prior exposure to TBHQ before an innate immune response in sentinel macrophages. However, it would also be important to examine the reverse order of exposure, as well as simultaneous exposures. At least under in vitro experimental conditions, the NF-κB p65 subunit competes with Nrf2 for co-factors leading to the inactivation of Nrf2, and Nrf2 can induce A20 protein, leading to an inhibition IKK-β phosphorylation [63].

While these findings provide important insights into TBHQ effects on macrophage activation, several limitations should be considered in their interpretation. Firstly, RAW 264.7 cells are an immortalized cell line that can accumulate genetic and metabolic aberrations and are only recommended for use up to 30 passages [64]. Primary macrophages, such as bone-marrow-derived, peritoneal, and/or tissue resident, should be evaluated to confirm translatability, though anatomically distinct macrophages have very different gene and protein expression profiles [65,66]. Additionally, media concentrations may not reflect intracellular or tissue-relevant levels, as TBHQ can rapidly oxidize to TBBQ [67,68]. Measuring intracellular parent/metabolite levels may further improve conclusions. In this study, initial functional data were captured at 24 h, while transcriptomics were evaluated at 12 h, though expression of COX-2 and ACOD1 proteins, and iNOS activities at 12 h validated the RNAseq data. Pharmacologic or genetic targeting of Nrf2, Keap1, HO-1, or glutathione synthesis pathways would help validate whether these pathways are required for TBHQ’s observed phenotype. Lastly, given the relative underuse of PIC in immunological/toxicological models compared to LPS, future studies should continue to explore its utility alongside traditional bacterial stimulants.

## 5. Conclusions

This study examined the immunomodulatory effects of tert-butylhydroquinone (TBHQ) on murine RAW 264.7 macrophages, highlighting its potential to significantly influence inflammatory responses during exposure to lipopolysaccharide (LPS) and polyinosinic-polycytidylic acid (poly I:C, PIC). TBHQ was evaluated at concentrations of 0.1, 1, and 5 µM. Key findings from this study include: 1. TBHQ modulates inflammatory responses. At the highest non-cytotoxic dose (5 µM), TBHQ reduced nitric oxide production and interleukin-10 (IL-10) secretion, while lower concentrations (0.1 and 1 µM) enhanced both phagocytic activity and IL-6 secretion, underscoring its nuanced role in immune regulation. 2. TBHQ alters inflammatory signaling transcriptomic profiling: through functional assays and transcriptomic analyses, TBHQ was shown to significantly downregulate proinflammatory pathways. Critical genes involved in cytokine and chemokine production, such as *Cxcl2*, *Ccl2*, and *Il6*, were suppressed. Additionally, essential components of NF-κB signaling (including *Nfkbia*, *Nfkb1*, and *Ikbke*) exhibited considerable downregulation, suggesting a large impact on inflammatory signaling cascades. 3. TBHQ enhances antioxidant pathways: TBHQ’s effects were marked by the activation of potent antioxidant pathways, particularly those related to glutathione metabolism and xenobiotic processing. There was a notable increase in transcript levels for metabolic genes like *Cbr3*, *Adh7*, and *Gstp1/3*, indicating a shift toward enhanced cellular defense mechanisms. In conclusion, the findings demonstrate that TBHQ’s influence on macrophage activation and its capacity to modulate inflammatory signaling are primarily driven by the induction of Nrf2-related antioxidant pathways. This highlights the need for a critical reassessment of TBHQ’s use in food products, considering its implications for immune function and overall human health, as well as ecological risks associated with its widespread application.

## Figures and Tables

**Table 1:** Differential gene expression (DGE) of key genes involved in inflammation (KEGG pathways mmu04668, mmu04060, mmu04657, and mmu04061) and cellular metabolism (KEGG pathways mmu00480 and mmu00980) derived from KEGG pathway enrichment analysis. Values represent the log2FC relative to the control condition. All values listed were statistically significant (*p* value < 0.05).

Gene Name	Gene ID	Genbank NO.	Symbol	TBHQ *	LPS	LPS + TBHQ	PIC	PIC + TBHQ
*Inflammation*								
Tumor necrosis factor	21926	NM_013693.3	*Tnf*	+0.69	+2.05	+1.81	+3.16	+2.32
Interleukin 6	16193	NM_031168.2	*Il6*	−1.44	+3.14	+1.80	+5.32	+3.47
C-C motif chemokine ligand 12	20293	NM_011331.3	*Ccl12*	-	+5.02	-	+5.88	+2.67
C-C motif chemokine ligand 2	20296	NM_011333.3	*Ccl2*	+0.55	+4.00	+2.97	+4.80	+3.82
C-X-C motif chemokine ligand 2	20310	NM_009140.2	*Cxcl2*	+0.93	+3.91	+3.70	+4.82	+4.06
C-X-C motif chemokine ligand 3	330122	NM_203320.3	*Cxcl3*	-	+6.76	+6.84	+8.53	+8.51
C-X-C motif chemokine ligand 10	15945	NM_021274.2	*Cxcl10*	+0.65	+0.68	−0.71	+3.41	+1.89
Nitric oxide synthase 2, inducible	18126	NM_010927.4	*Nos2*	-	+2.36	+1.89	+2.90	+2.32
Prostaglandin-endoperoxide synthase 2	19225	NM_011198.5	*Ptgs2*	+1.36	+4.26	+3.57	+5.82	+4.70
Aconitate decarboxylase 1	16365	NM_008392.1	*Acod1*	+0.88	+4.08	+3.95	+5.24	+4.91
Nuclear factor of kappa light polypeptide gene enhancer in B cells 1, p105	18033	NM_008689.3	*Nfkb1*	+0.27	+0.57	+0.32	+1.27	+0.79
Nuclear factor of kappa light polypeptide gene enhancer in B cells inhibitor, alpha	18035	NM_010907.2	*Nfkbia*	+0.41	+1.36	+0.99	+2.25	+1.61
Inhibitor of kappaB kinase epsilon	56489	NM_019777.3	*Ikbke*	-	+1.57	+1.43	+2.29	+1.94
Interleukin 1 beta	16176	NM_008361.4	*Il1b*	-	+10.02	+7.03	+11.54	+8.33
Colony stimulating factor 3	12985	NM_009971.1	*Csf3*	+2.22	+8.07	+7.76	+8.91	+8.19
Matrix metallopeptidase 9	17395	NM_013599.5	*Mmp9*	+0.61	+2.56	+2.49	+2.95	+2.81
*Cellular Metabolism*								
Glutathione S-transferase, pi 1	14870	NM_013541.1	*Gstp1*	-	+0.64	+1.32	-	+1.09
Glutathione S-transferase pi 3	225884	NM_144869.4	*Gstp3*	-	+5.72	+5.20	+6.68	+5.16
Glutathion S-transferase, alpha 3	14859	NM_010365.5	*Gsta3*	-	-	+2.47	-	+2.68
Carbonyl reductase 3	109857	NM_173047.3	*Cbr3*	+1.46	-	+1.27	-	+1.17
Alcohol dehydrogenase 7 (class IV), mu or sigma polypeptide	11529	NM_009626.4	*Adh7*	−0.67	+0.45	+1.16	-	+1.00
Ornithine decarboxylase, structural 1	18263	NM_013614.3	*Odc1*	+0.29	+1.93	+1.55	+2.59	+2.09
Glucose-6-phosphate dehydrogenase X-linked	14381	NM_008062.3	*G6pdx*	+0.67	+0.71	+1.12	+0.80	+1.17
Glutamate-cysteine ligase, modifer subunit	14630	NM_008129.4	*Gclm*	+1.82	+0.94	+1.92	+0.73	+1.83
Chac, cation transport regulator 1	69065	NM_026929.4	*Chac1*	-	+1.39	+1.26	+1.35	+1.33
Heme oxygenase 1	15368	NM_010442.2	*Hmox1*	+2.10	+2.34	+2.04	+2.58	+2.41

* TBHQ treatment was assessed at 4 h to determine antioxidant effects; all other treatments adhered to the 12 h study design.

## Data Availability

Data is contained within the article. Sequencing data can be accessed at the GEO data repository (GSE 307170).

## Supplementary Materials

The following supporting information can be downloaded at: https://www.mdpi.com/article/10.3390/toxics13100883/s1, Figure S1: Cell viability following TBHQ treatment of RAW 264.7 macrophages using the MTT assay. Cells were treated with serial dilutions of TBHQ for 24 h. TBHQ at 5 µM and below were not cytotoxic. Data is expressed as percentage of control optical densities, and presented as the mean +/− SE. The assays were carried out in triplicate; Figure S2: Principal Component Analysis (PCA) plot shows close clustering of all sample replicates, indicating low technical variation; Figure S3: Extended dot plot of all 42 upregulated Kyoto Encyclopedia of Genes and Genomes (KEGG) pathways, of which at least one of the treatment groups is enriched. All pathways are statistically significant (*p* ≤ 0.05), and the color of the dots represent the FDR value, where darker reds are more statistically significant. The size of the dot represents the fold enrichment, where larger dots are proportional to a larger fold-enrichment value; Figure S4: Heatmaps of the full Kyoto Encyclopedia of Genes and Genomes (KEGG) pathways selected for further analysis in the body of the paper. (A) TNF signaling pathway (mmu04668), including 118 genes. (B) Cytokine cytokine receptor interaction (mmu04060), including 293 genes. (C) IL-17 signaling pathway (mmu04657), including 93 genes. (D) Viral protein interaction with cytokine and cytokine receptor (mmu04061), including 95 genes. (E) Glutathione metabolism (mmu00480), including 72 genes. (F) Metabolism of xenobiotics by cytochrome P450 (mmu00980), including 75 genes. A–D represents the four most biologically significant upregulated KEGG pathways across treatments related to inflammation. Heatmaps E–F are the two most biologically significant and upregulated KEGG pathways related to cellular metabolism, specifically enriched in TBHQ treatments; Table S1: Amino acid sequences of predicted most immunogenic ACOD1 peptides (underlined) from representative vertebrates (F, fish; Rp, reptile; Avian, Av; Mammal, M) demonstrating a highly conserved core ACOD1 epitope (TFYGHWRKPL). Mice were immunized with synthetic mouse ACOD1 peptide conjugated to KLH at the C-terminus cysteine (C) and resulting hybridomas screened against the unconjugated peptide by ELISA. Positive hybridomas were subsequently re-screened by ELISA against the core sequence (TFYGHWRKPL) to obtain mAb AW-12 with reactivity across vertebrate taxa.

## References

[1] Godfray H.C.J., Beddington J.R., Crute I.R., Haddad L., Lawrence D., Muir J.F., Pretty J., Robinson S., Thomas S.M., Toulmin C. (2010). Food Security: The Challenge of Feeding 9 Billion People. Science.

[2] Fung F., Wang H.-S., Menon S. (2018). Food safety in the 21st century. Biomed. J..

[3] King T., Cole M., Farber J.M., Eisenbrand G., Zabaras D., Fox E.M., Hill J.P. (2017). Food safety for food security: Relationship between global megatrends and developments in food safety. Trends Food Sci. Technol..

[4] Pressman P., Clemens R., Hayes W., Reddy C. (2017). Food additive safety: A review of toxicologic and regulatory issues. Toxicol. Res. Appl..

[5] Shahidi F., Ambigaipalan P. (2015). Phenolics and polyphenolics in foods, beverages and spices: Antioxidant activity and health effects—A review. J. Funct. Foods.

[6] Esazadeh K., Ezzati Nazhad Dolatabadi J., Andishmand H., Mohammadzadeh-Aghdash H., Mahmoudpour M., Naemi Kermanshahi M., Roosta Y. (2024). Cytotoxic and genotoxic effects of tert-butylhydroquinone, butylated hydroxyanisole and propyl gallate as synthetic food antioxidants. Food Sci. Nutr..

[7] Khezerlou A., Akhlaghi A.P., Alizadeh A.M., Dehghan P., Maleki P. (2022). Alarming impact of the excessive use of tert-butylhydroquinone in food products: A narrative review. Toxicol. Rep..

[8] Liu X., Zheng Z., Liu Y. (2025). Lipophilic antioxidants in edible oils: Mechanisms, applications and interactions. Food Res. Int..

[9] Zhou J., Xiong Y., Shi Y. (2016). Antioxidant Consumption Kinetics and Shelf-Life Prediction for Biodiesel Stabilized with Antioxidants Using the Rancimat Method. Energy Fuels.

[10] Li Z.J., Yang F.J., Yang L., Zu Y.G. (2018). Comparison of the antioxidant effects of carnosic acid and synthetic antioxidants on tara seed oil. Chem. Cent. J..

[11] Gharavi N., Haggarty S., El-Kadi A.O. (2007). Chemoprotective and carcinogenic effects of tert-butylhydroquinone and its metabolites. Curr. Drug Metab..

[12] World Health Organization (1999). Safety Evaluation of Certain Food Additive and Contaminants.

[13] Botterweck A.A.M., Verhagen H., Goldbohm R.A., Kleinjans J., van den Brandt P.A. (2000). Intake of butylated hydroxyanisole and butylated hydroxytoluene and stomach cancer risk: Results from analyses in The Netherlands Cohort Study. Food Chem. Toxicol..

[14] Engelhardt J.A., Athanassiadis I., Leonards P.E.G., Weiss J.M. (2025). Multi-target analysis of synthetic phenolic compounds in human blood. Talanta.

[15] Wang S., Zhang H., Shi X., Zhang Q., Ren Y., Chen J., Wang H., Wang W., Wang Q. (2025). The reaction laws and toxicity effects of synthetic phenolic antioxidants (SPAs) in wastewater and atmosphere: A theoretical investigation. Process Saf. Environ. Prot..

[16] Australian Industrial Chemicals Introduction Scheme (2022). Butylated Hydroxyanisole and Related Antioxidants.

[17] Hoang N.M.H., Park K. (2024). Applications of Tert-Butyl-Phenolic Antioxidants in Consumer Products and Their Potential Toxicities in Humans. Toxics.

[18] van Esch G.J. (1986). Toxicology of tert-Butylhydroquinone (TBHQ). Food Chem. Toxicol..

[19] National Toxicology Program (1997). Toxicology and Carcinogenesis Studies of t-Butylhydroquinone in F344/N Rats and B6C3F1 Mice.

[20] Hahn M.E., McArthur A.G., Karchner S.I., Franks D.G., Jenny M.J., Timme-Laragy A.R., Stegeman J.J., Woodin B.R., Cipriano M.J., Linney E. (2014). The transcriptional response to oxidative stress during vertebrate development: Effects of tert-butylhydroquinone and 2,3,7,8-tetrachlorodibenzo-p-dioxin. PLoS ONE.

[21] Baran A., Yildirim S., Ghosigharehaghaji A., Bolat İ., Sulukan E., Ceyhun S.B. (2021). An approach to evaluating the potential teratogenic and neurotoxic mechanism of BHA based on apoptosis induced by oxidative stress in zebrafish embryo (*Danio rerio*). Hum. Exp. Toxicol..

[22] Leonard E.R., Marques E.S., Roy M.A., Conlin S.M., Ranjan R., Timme-Laragy A.R. (2023). Dietary exposure to the food preservative tert-Butylhydroquinone (tBHQ) impairs zebrafish (*Danio rerio*) survival, growth, organ development, and gene expression in Nrf2a-dependent and independent ways. Food Chem. Toxicol..

[23] Yang X., Sun Z., Wang W., Zhou Q., Shi G., Wei F., Jiang G. (2018). Developmental toxicity of synthetic phenolic antioxidants to the early life stage of zebrafish. Sci. Total Environ..

[24] Wufuer R., Fan Z., Liu K., Zhang Y. (2021). Differential Yet Integral Contributions of Nrf1 and Nrf2 in the Human HepG2 Cells on Antioxidant Cytoprotective Response against Tert-Butylhydroquinone as a Pro-Oxidative Stressor. Antioxidants.

[25] Zagorski J.W., Turley A.E., Dover H.E., VanDenBerg K.R., Compton J.R., Rockwell C.E. (2013). The Nrf2 Activator, tBHQ, Differentially Affects Early Events Following Stimulation of Jurkat Cells. Toxicol. Sci..

[26] Lazaro I., Lopez-Sanz L., Bernal S., Oguiza A., Recio C., Melgar A., Jimenez-Castilla L., Egido J., Madrigal-Matute J., Gomez-Guerrero C. (2018). Nrf2 Activation Provides Atheroprotection in Diabetic Mice Through Concerted Upregulation of Antioxidant, Anti-inflammatory, and Autophagy Mechanisms. Front. Pharmacol..

[27] Imhoff B.R., Hansen J.M. (2010). Tert-butylhydroquinone induces mitochondrial oxidative stress causing Nrf2 activation. Cell Biol. Toxicol..

[28] Rockwell C.E., Zhang M., Fields P.E., Klaassen C.D. (2012). Th2 Skewing by Activation of Nrf2 in CD4+ T Cells. J. Immunol..

[29] Jin Y., Boss A.P., Bursley J.K., Wilson C., Gangur V., Rockwell C.E. (2025). The transcription factor Nrf2 links Th2-mediated experimental allergy to food preservatives. Front. Immunol..

[30] Boss A.P., Freeborn R.A., Duriancik D.M., Kennedy R.C., Gardner E.M., Rockwell C.E. (2018). The Nrf2 activator tBHQ inhibits the activation of primary murine natural killer cells. Food Chem. Toxicol..

[31] Gharavi N., El-Kadi A.O. (2005). tert-Butylhydroquinone is a novel aryl hydrocarbon receptor ligand. Drug Metab. Dispos..

[32] Zhao Y.-L., Zhao W., Liu M., Liu L., Wang Y. (2020). TBHQ-Overview of Multiple Mechanisms against Oxidative Stress for Attenuating Methamphetamine-Induced Neurotoxicity. Oxidative Med. Cell. Longev..

[33] Zhang D.D., Hannink M. (2003). Distinct Cysteine Residues in Keap1 Are Required for Keap1-Dependent Ubiquitination of Nrf2 and for Stabilization of Nrf2 by Chemopreventive Agents and Oxidative Stress. Mol. Cell. Biol..

[34] Zagorski J.W., Turley A.E., Freeborn R.A., VanDenBerg K.R., Dover H.E., Kardell B.R., Liby K.T., Rockwell C.E. (2018). Differential effects of the Nrf2 activators tBHQ and CDDO-Im on the early events of T cell activation. Biochem. Pharmacol..

[35] Medzhitov R. (2021). The spectrum of inflammatory responses. Science.

[36] Hirayama D., Iida T., Nakase H. (2017). The Phagocytic Function of Macrophage-Enforcing Innate Immunity and Tissue Homeostasis. Int. J. Mol. Sci..

[37] Mantovani A., Sica A., Sozzani S., Allavena P., Vecchi A., Locati M. (2004). The chemokine system in diverse forms of macrophage activation and polarization. Trends Immunol..

[38] Janeway C.A. (2001). How the immune system protects the host from infection. Microbes Infect..

[39] Moratilla-Rivera I., Sánchez M., Valdés-González J.A., Gómez-Serranillos M.P. (2023). Natural Products as Modulators of Nrf2 Signaling Pathway in Neuroprotection. Int. J. Mol. Sci..

[40] Pouremamali F., Pouremamali A., Dadashpour M., Soozangar N., Jeddi F. (2022). An update of Nrf2 activators and inhibitors in cancer prevention/promotion. Cell Commun. Signal..

[41] Babcock A.S., Anderson A.L., Rice C.D. (2013). Indirubin-3′-(2,3 dihydroxypropyl)-oximether (E804) is a potent modulator of LPS-stimulated macrophage functions. Toxicol. Appl. Pharmacol..

[42] Dietert R.R., Hotchkiss J.H., Austic R.E., Sung Y.-J., Burleson G.R., Dean J.H., Munson A.E. (1995). Production of reactive nitrogen intermediates by macrophages. Methods in Immunotoxicology.

[43] Nguyen T., Sherratt P.J., Nioi P., Yang C.S., Pickett C.B. (2005). Nrf2 Controls Constitutive and Inducible Expression of ARE-driven Genes through a Dynamic Pathway Involving Nucleocytoplasmic Shuttling by Keap1*. J. Biol. Chem..

[44] Li C.-Q., Kim M.Y., Godoy L.C., Thiantanawat A., Trudel L.J., Wogan G.N. (2009). Nitric oxide activation of Keap1/Nrf2 signaling in human colon carcinoma cells. Proc. Natl. Acad. Sci. USA.

[45] Anderson A.L., Dubanksy B.D., Wilson L.B., Tanguay R.L., Rice C.D. (2022). Development and Applications of a Zebrafish (*Danio rerio*) CYP1A-Targeted Monoclonal Antibody (CRC4) with Reactivity across Vertebrate Taxa: Evidence for a Conserved CYP1A Epitope. Toxics.

[46] Margiotta A.L., Bain L.J., Rice C.D. (2017). Expression of the Major Vault Protein (MVP) and Cellular Vault Particles in Fish. Anat. Rec..

[47] Schneider C.A., Rasband W.S., Eliceiri K.W. (2012). NIH Image to ImageJ: 25 years of image analysis. Nat. Methods.

[48] Li W., Kong A.N. (2009). Molecular mechanisms of Nrf2-mediated antioxidant response. Mol. Carcinog..

[49] Li R., Jia Z., Zhu H. (2019). Regulation of Nrf2 Signaling. React. Oxyg. Species.

[50] Tonelli C., Chio I.I.C., Tuveson D.A. (2018). Transcriptional Regulation by Nrf2. Antioxid. Redox Signal..

[51] Ngo V., Duennwald M.L. (2022). Nrf2 and Oxidative Stress: A General Overview of Mechanisms and Implications in Human Disease. Antioxidants.

[52] Trask O.J., Markossian S., Grossman A., Baskir H., Arkin M., Auld D., Austin C., Baell J., Brimacombe K., Chung T.D.Y., Coussens N.P. (2012). Translocation Assay Development and Validation for High Content Screening.

[53] Reimer T., Brcic M., Schweizer M., Jungi T.W. (2008). poly(I:C) and LPS induce distinct IRF3 and NF-κB signaling during type-I IFN and TNF responses in human macrophages. J. Leukoc. Biol..

[54] Kawai T., Akira S. (2008). Toll-like receptor and RIG-I-like receptor signaling. Ann. N. Y. Acad. Sci..

[55] Alexopoulou L., Holt A.C., Medzhitov R., Flavell R.A. (2001). Recognition of double-stranded RNA and activation of NF-kappaB by Toll-like receptor 3. Nature.

[56] Yang Z.-X., Guo D.-Y., Shen L.-Z., Mao G.-X., Dai J.-H., Chen S.-S., Yan J. (2019). Transcriptome profiling of poly(I:C)-induced RAW 264.7 mouse macrophages in response to panaxadiol. Biologia.

[57] Kim Y.-J., Kim H.-J., Lee J.Y., Kim D.-H., Kang M.S., Park W. (2018). Anti-Inflammatory Effect of Baicalein on Polyinosinic–Polycytidylic Acid-Induced RAW 264.7 Mouse Macrophages. Viruses.

[58] Palmieri E.M., McGinity C., Wink D.A., McVicar D.W. (2020). Nitric Oxide in Macrophage Immunometabolism: Hiding in Plain Sight. Metabolites.

[59] Pradhan P., Vijayan V., Liu B., Martinez-Delgado B., Matamala N., Nikolin C., Greite R., DeLuca D.S., Janciauskiene S., Motterlini R. (2024). Distinct metabolic responses to heme in inflammatory human and mouse macrophages—Role of nitric oxide. Redox Biol..

[60] Ye Q., Gao Z., Yan H., Zhang H., Meng X., Xiao H. (2025). Transcriptomic analysis reveals significant changes in gene expression and a potential mitochondria-mediated apoptotic pathway in murine RAW 264.7 macrophages exposed to tert-butylquinone. Food Biosci..

[61] O’Neill L.A.J., Artyomov M.N. (2019). Itaconate: The poster child of metabolic reprogramming in macrophage function. Nat. Rev. Immunol..

[62] Wu R., Chen F., Wang N., Tang D., Kang R. (2020). ACOD1 in immunometabolism and disease. Cell. Mol. Immunol..

[63] Gao W., Guo L., Yang Y., Wang Y., Xia S., Gong H., Zhang B.-K., Yan M. (2022). Dissecting the Crosstalk Between Nrf2 and NF-κB Response Pathways in Drug-Induced Toxicity. Front. Cell Dev. Biol..

[64] Taciak B., Bialasek M., Braniewska A., Sas Z., Sawicka P., Kiraga L., Rygiel T., Krol M. (2018). Evaluation of phenotypic and functional stability of RAW 264.7 cell line through serial passages. PLoS ONE.

[65] Gordon S., Plüddemann A. (2017). Tissue macrophages: Heterogeneity and functions. BMC Biol..

[66] Guan F., Wang R., Yi Z., Luo P., Liu W., Xie Y., Liu Z., Xia Z., Zhang H., Cheng Q. (2025). Tissue macrophages: Origin, heterogenity, biological functions, diseases and therapeutic targets. Signal Transduct. Target. Ther..

[67] Ye Q., Meng X., Jiang L. (2018). Identification and assessment of residual levels of the main oxidation product of tert-butylhydroquinone in frying oils after heating and its cytotoxicity to RAW 264.7 cells. Food Chem..

[68] Okubo T., Yokoyama Y., Kano K., Kano I. (2003). Cell death induced by the phenolic antioxidant tert-butylhydroquinone and its metabolite tert-butylquinone in human monocytic leukemia U937 cells. Food Chem. Toxicol..

