# Supplementary material for: Tert-Butylhydroquinone (TBHQ) Suppresses LPS- and Poly (I:C)-Induced RAW 264.7 Macrophage Activation Through Reduced NF-κB/Type 1 Interferon and Enhanced Antioxidant-Related Pathways
Source: Toxics. 2025 Oct 16;13(10):883. doi: 10.3390/toxics13100883 (PMC12568040; doi:10.3390/toxics13100883)
Supplement: Supplementary file 1 [file toxics-13-00883-s001.zip › toxics-3887691-supplementary.pdf]

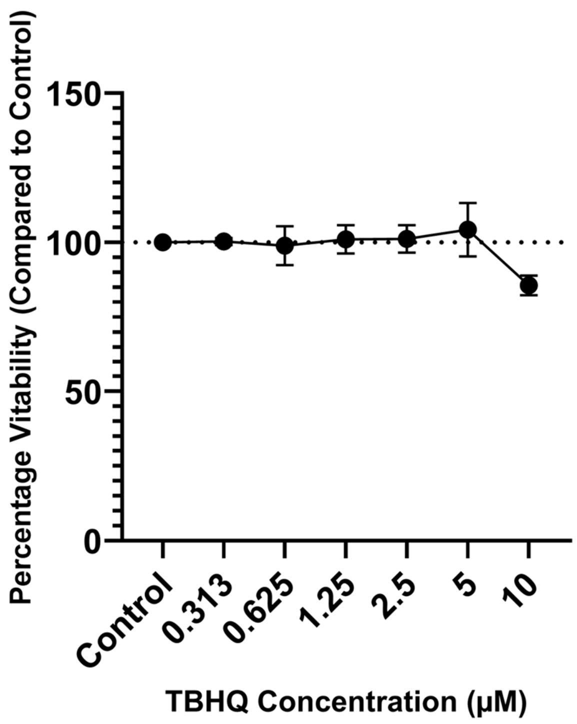

Supplemental Figure 1. Cell viability following TBHQ treatment of RAW 264.7 macrophages using the MTT assay. Cells were treated with serial dilutions of TBHQ for 24 h. TBHQ at 5 μM and below were not cytotoxic. Data is expressed as percentage of control optical densities, and presented as the mean +/- SE. The assays were carried out in triplicate.

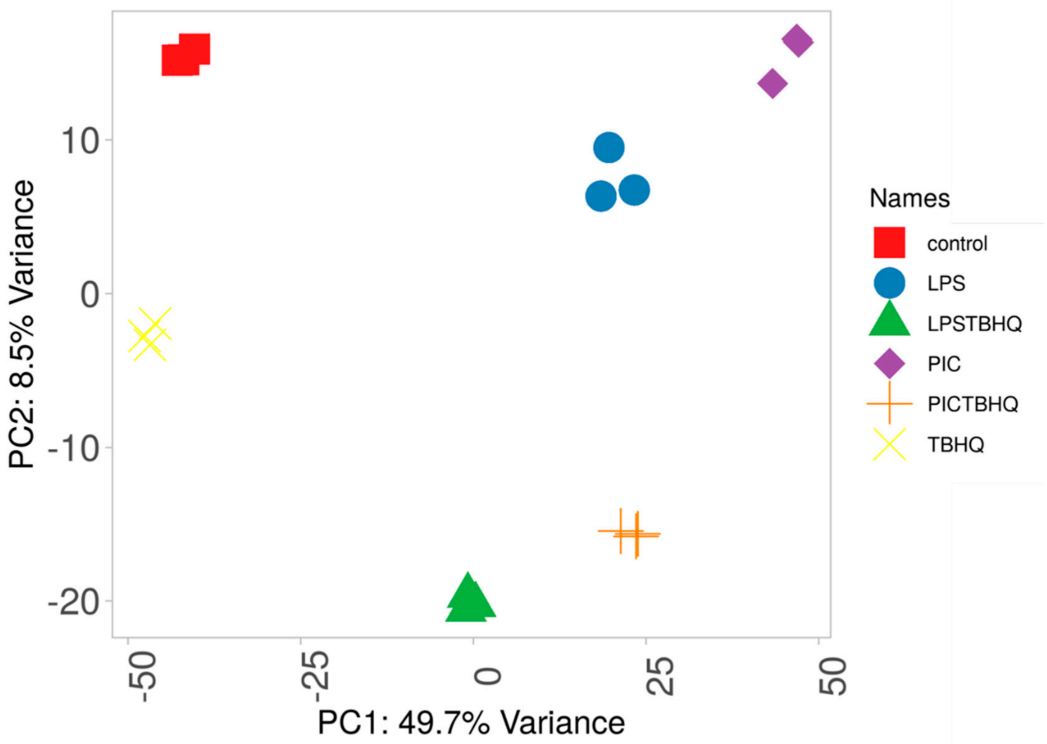

Supplemental Figure 2. Principal Component Analysis (PCA) plot shows close clustering of all sample replicates, indicating low technical variation.

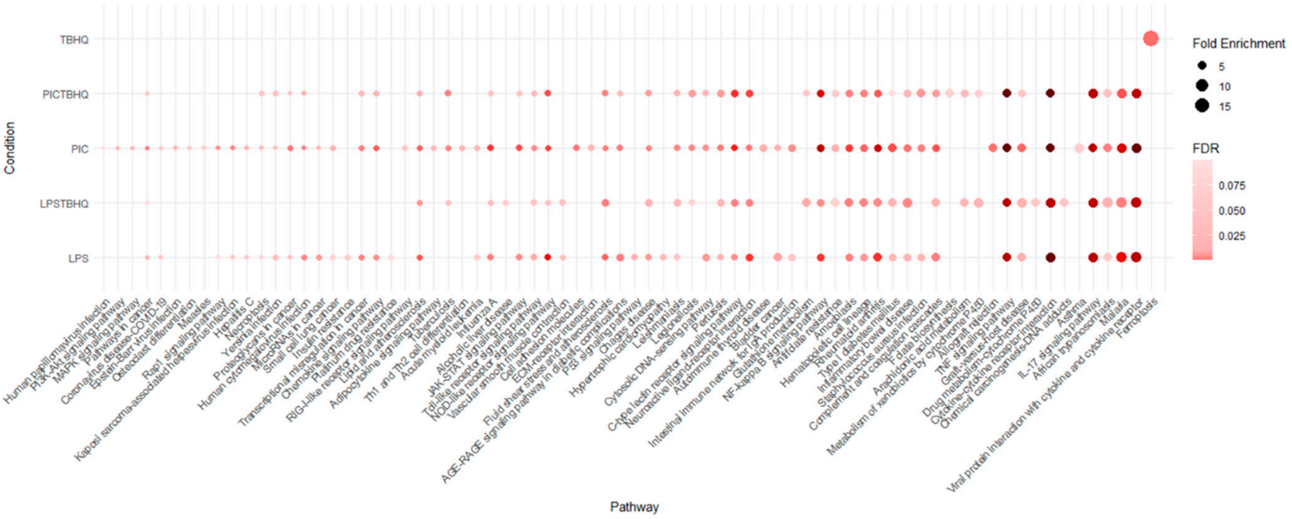

Supplemental Figure 3. Extended dot plot of all 42 upregulated Kyoto Encyclopedia of Genes and Genomes (KEGG) pathways, of which at least one of the treatment groups is enriched. All pathways are statistically significant (P≤0.05), and the color of the dots represent the FDR value, where darker reds are more statistically significant. The size of the dot represents the fold enrichment, where larger dots are proportional to a larger fold-enrichment value.

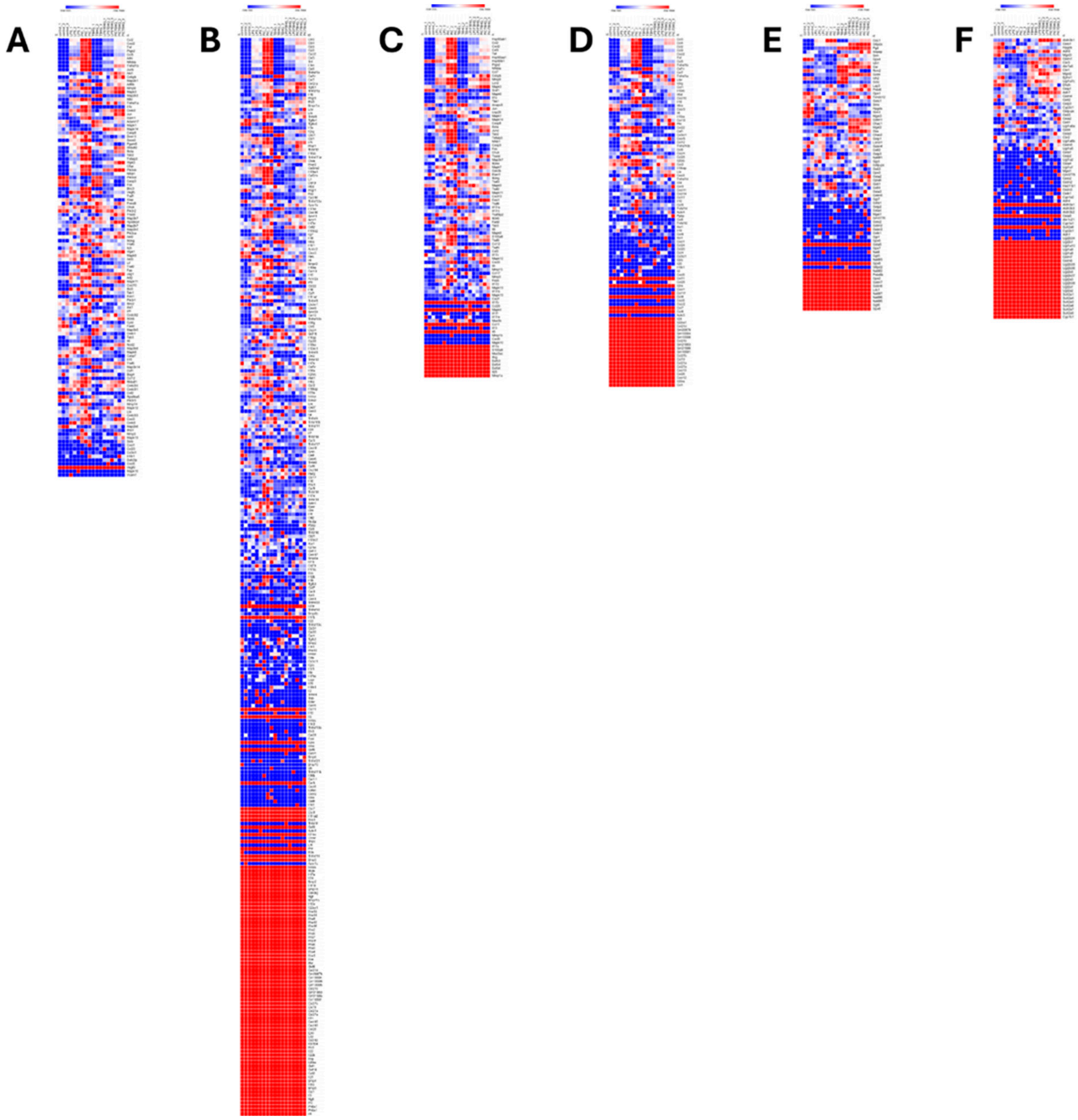

Supplemental Figure 4. Heatmaps of the full Kyoto Encyclopedia of Genes and Genomes (KEGG) pathways selected for further analysis in the body of the paper. A) TNF signaling pathway (mmu04668), including 118 genes. B) Cytokine cytokine receptor interaction (mmu04060), including 293 genes. C) IL-17 signaling pathway (mmu04657), including 93 genes. D) Viral protein interaction with cytokine and cytokine receptor (mmu04061), including 95 genes. E) Glutathione metabolism (mmu00480), including 72 genes. F) Metabolism of xenobiotics by cytochrome P450 (mmu00980),

including 75 genes. A-D represents the four most biologically significant upregulated KEGG pathways across treatments related to inflammation. Heatmaps E-F are the two most biologically significant and upregulated KEGG pathways related to cellular metabolism, specifically enriched in TBHQ treatments.

Supplemental Methods: Generation of mAb AW-12

Supplemental Materials and Methods

Development of a pan-vertebrate-specific antibody against ACOD1: Immunogen design

Generation of the anti-ACOD1 monoclonal antibody began with the design of an immunogenic synthetic peptide. The sequence was chosen based on Hopp–Woods hydrophilicity profiles (https://web.expasy.org/protscale/), NIH-Ab-designer algorithms (https://esbl.nhlbi.nih.gov/AbDesigner/), Immune Epitope Data Base algorithms (https://www.iedb.org), and peptide solubility (http://pepcalc.com) algorithms. The final peptide sequence from full length mouse ACOD1 (Gene Bank: NP_032418.1) was then checked for possible negative internal amino acid interactions. These sites and algorithms were last visited on January 10, 2024. The synthetic peptide was synthesized and a portion conjugated to keyhole limpet hemocyanin (KLH) (Innovogen, Sweden) at the n-terminus of the peptide using a cysteine bridge. Unconjugated peptide was used to screen anti-sera and primary hybridomas prior to cloning by limiting dilution. The synthetic peptide was also chosen based on a conserved epitope shared across vertebrate phyla (Table 1 below).

Development and characterization of a monoclonal antibody against ACOD1

Six-week-old female Balb/c mice (Charles River) were used for immunizations and housed at Godley Snell Animal Facility, a Clemson University IACUC approved facility under an IACUC-approved AUP protocol. Mice were given a subcutaneous (s.c.) injection with 100 µg of immunogen in 0.9% saline containing TiterMax 21 Gold® adjuvant on day 1. Two weeks later mice were given a booster immunization using Freund's incomplete adjuvant. Additionally, further booster immunizations (s.c.) were administered without adjuvant in 21-day intervals, and the final immunization was given intraperitoneally. Five days following the final immunization, mice were euthanized via slow lethal CO_2 hypoxia, bled my cardiac puncture to collect polyclonal antisera, and their spleens were removed via aseptic technique. Spleen cells were fused with Sp2/m1L6 myeloma cells (ATCC # CRL-2016) using ClonaCell™-HYPEG (StemCell Technologies, #03806). Screening and cloning of respective hybridomas was done following previously published methods [1,2]. Primary hybridomas were cultured in DMEM supplemented with 10% FBS, 20 mM HEPES, 10 mM L-glutamine, 100 µg/mL penicillin, 100 µg/mL streptomycin, 110 µg/mL sodium pyruvate, 1% non-essential amino acids (from a 100× stock), 4.5 g/L glucose, 10 µg/mL gentamycin, and 5 µg/mL nystatin.

Primary hybridoma supernatants were then screened by ELISA for reactivity against the unconjugated target polypeptide. The resulting antibodies against the peptide included polyclonal anti-sera, which was subjected to protein-A/G purification steps to obtain purified IgG antibody, and several primary hybridoma supernatants. One primary hybridoma (mAb AW-12) was subsequently cloned and the isotype

determined using a Mouse Rapid-Isotype Kit® (Fisher Scientific) as an IgG_{2a} κ immunoglobulin.

Table 1. Amino acid sequences of predicted most immunogenic ACOD1 peptides (underlined) from representative vertebrates (F, fish; Rp, reptile; Avian, Av; Mammal, M) demonstrating a highly conserved core ACOD1 epitope (TFYGHWRKPL). Mice were immunized with synthetic mouse ACOD1 peptide conjugated to KLH at the C-terminus cysteine (C) and resulting hybridomas screened against the unconjugated peptide by ELISA. Positive hybridomas were subsequently re-screened by ELISA against the core sequence (TFYGHWRKPL) to obtain mAb AW-12 with reactivity across vertebrate taxa.

ACOD1 amino acid sequence	Species	Accession number
DTFYGHWRKPLSQE	Mouse, *M. musculus (m)*	NP_032418.1
DTFYGHWRKPLSQE	Human, *H. sapiens (m)*	NP_001245335.1
DTFYGHWRKPLSQE	Rat, *R. norvegicus (m)*	NP_001100752.1
DTFYGHWRKPLSQE	Rainbow trout, *O. mykiss (f)*	XP_021434284.2
NTFYGHWRKPLSQE	Channel catfish, *I. punctatus (f)*	XP_017326109.1
DTFYGHWRKPLSQE	Zebrafish, *D. rerio (f)*	NP_001119928.1
NTFYGHWRKPLSQQ	Mummichog, *F. heteroclitus (f)*	XP_012719711.2
NTFYGHWRKPLSHE	Fathead minnow, *P. promelas (f)*	XP_039524869.1
DTFYGHWRKPLSKE	Clawed frog, *X. trpicalis (am)*	XP_002931983.3
NTFYGHWRKPLTKG	Chicken, *G. gallus (av)*	XP_015130858.1
DTFYGHWRKPLSTD	Green sea turtle, *C. midas (r)*	XP_007058232.1
TFYGHWRKPL	Conserved ACOD1 epitope	All the above

References

1. Margiotta, A.L.; Bain, L.J.; Rice, C.D. Expression of the Major Vault Protein (MVP) and Cellular Vault Particles in Fish. *The Anatomical Record* **2017**, *300*, 1981-1992, doi:10.1002/ar.23645.
2. Anderson, A.L.; Dubanksy, B.D.; Wilson, L.B.; Tanguay, R.L.; Rice, C.D. Development and Applications of a Zebrafish (Danio rerio) CYP1A-Targeted Monoclonal Antibody (CRC4) with Reactivity across Vertebrate Taxa: Evidence for a Conserved CYP1A Epitope. *Toxics* **2022**, *10*, 404.
